# A Comparison of Digital Gene Expression Profiling and Methyl DNA Immunoprecipitation as Methods for Gene Discovery in Honeybee (*Apis mellifera*) Behavioural Genomic Analyses

**DOI:** 10.1371/journal.pone.0073628

**Published:** 2013-09-09

**Authors:** Cui Guan, Andrew B. Barron, Xu Jiang He, Zi Long Wang, Wei Yu Yan, Zhi Jiang Zeng

**Affiliations:** 1 Honeybee Research Institute, Jiangxi Agricultural University, Nanchang, Jiangxi, P.R. of China; 2 Department of Biological Sciences, Macquarie University, North Ryde, New South Wales, Australia; CNRS, France

## Abstract

The honey bee has a well-organized system of division of labour among workers. Workers typically progress through a series of discrete behavioural castes as they age, and this has become an important case study for exploring how dynamic changes in gene expression can influence behaviour. Here we applied both digital gene expression analysis and methyl DNA immunoprecipitation analysis to nurse, forager and reverted nurse bees (nurses that have returned to the nursing state after a period spent foraging) from the same colony in order to compare the outcomes of these different forms of genomic analysis. A total of 874 and 710 significantly differentially expressed genes were identified in forager/nurse and reverted nurse/forager comparisons respectively. Of these, 229 genes exhibited reversed directions of gene expression differences between the forager/nurse and reverted nurse/forager comparisons. Using methyl-DNA immunoprecipitation combined with high-throughput sequencing (MeDIP-seq) we identified 366 and 442 significantly differentially methylated genes in forager/nurse and reverted nurse/forager comparisons respectively. Of these, 165 genes were identified as differentially methylated in both comparisons. However, very few genes were identified as both differentially expressed and differentially methylated in our comparisons of nurses and foragers. These findings confirm that changes in both gene expression and DNA methylation are involved in the nurse and forager behavioural castes, but the different analytical methods reveal quite distinct sets of candidate genes.

## Introduction

The honey bee has emerged as a key model system for research in behavioural genomics. A honey bee colony is a society of up to 50,000 closely related and sterile worker bees all descended from a single queen. Within this society there is a marked division of labour. Even though all worker bees are morphologically indistinguishable, different bees are behaviourally specialized to different roles. Workers change roles as they age in a predictable pattern, typically beginning life working on diverse tasks within the hive (particularly brood care), and working outside the hive as forager bees when they are older [1]. However there is also flexibility inherent to this system. In response to social feedback from within the colony the rate at which bees progress between different behavioural states can be accelerated, delayed or even reversed [2], [3]. This has provided a valuable natural and social model system with which to examine how genomic factors can interact with social stimuli to influence individual behavioural specialization.

Tools and techniques for honey bee genomic analyses are evolving rapidly. The greatest focus in honey bee behavioural genomics has been to compare nurse bees (bees sampled while feeding developing larvae) and forager bees (bees sampled collecting floral resources for their colony), since these are two stable, mutually exclusive and highly distinct behavioural classes. Early studies used a genomic microarray to compare brain gene expression profiles of these behavioural groups [4], and revealed that 39% of honey bee genes were dynamically regulated and significantly different in expression between nurses and foragers [4]. Even after controlling for age differences gene expression profiles between nurse and forager bees were so distinct that it was possible to predict behavioural state based purely on the genomic profile [4]. Later studies confirmed these core findings, and the dramatic extent to which changes in gene expression level are associated with changes in behaviour [5]–[7]. Comparative analyses with other bee species have drawn similar conclusions [8].

Continual improvements in the precision and economies of next-generation sequencing technologies have resulted in a shift away from reliance on microarray methods for gene expression profile analysis to the use of digital sequencing technologies [9], [10]. Digital sequencing methods have several advantages over microarray techniques, particularly in terms of absolute quantification of mRNA transcript abundance, and the greater ease of detection of splice variants [9], [10]. Digital gene expression (DGE) methods have also recently been used to compare gene expression profiles in nurse and forager honey bees [11], with largely comparable results to the earlier microarray analyses.

The discovery of a functional gene methylation system in honey bees [12] has triggered vigorous interest in the possible role of epigenetic regulation of the genome in the organization of social and behavioural specialization. The honey bee genome is sparsely methylated compared to mammalian genomes, and unlike mammals it is common to find methylated sites within the bodies of genes [13]. It seems clear that in honey bees methylation is involved in the regulation of alternative splicing as well as epigenetic control of gene expression [13]. The two different developmental pathways differentiating worker and queen phenotypes are epigenetically regulated [13], [14], and epigenetic processes are involved in bee learning and memory [15], and recently differences in genomic methylation profile were demonstrated between nurse and forager bees [16]. Some of these differences showed a highly dynamic relationship with behavioural state since some of the methylation differences observed between nurse and forager bees appeared to be reversed in samples comparing forager bees to reverted nurses (bees that had transitioned from foragers back to the nursing state) [16].

MeDIP-seq, which combines methylated DNA immunoprecipitation (MeDIP) with next-generation sequencing, has been used to describe the DNA methylome of several different species [17]–[20]. MeDIP-seq is considered more cost-effective than bisulphate sequencing, and a valid method for comparing relative differences in methylation between samples [21]–[23].

It is generally assumed that increased methylation of a gene will result in reduced expression of a gene, whereas decreased methylation will result in increased expression, but this has not been broadly tested in insects where methylation occurs within gene bodies as well as within the promoter regions. Further, methylation is just one of many possible mechanisms regulating gene expression. It is therefore interesting to explore the degree to which there is overlap between genes significantly differentially expressed and methylated between forager and nurse bees. This analysis would show to what extent the well-characterized differences in gene expression between these behavioural groups are associated with changes in methylation. The experiment presented here was designed to address this issue. Here we compared both gene expression differences and gene methylation differences using DGE and MeDIP techniques in nurses, foragers and reverted nurses from the same colony. Our aim was to assess both the comparability of analyses using these techniques with existing published comparisons, and also the degree of congruence between the gene lists generated from DGE and MeDIP techniques.

## Materials and Methods

### Insect

Honeybees (*Apis mellifera*) were maintained at the Honeybee Research Institute, Jiangxi Agricultural University, Nanchang, China (28.46°N, 115.49°E) using standard beekeeping techniques. All of the samples were collected from the same colony to minimize variation in genetic background of the sampled bees. Bees collected when feeding larvae were considered as nurses, while foragers were distinguished by the colored pollen loads in their corbiculae. All sampled bees were flash frozen in liquid nitrogen immediately after harvesting, and heads were stored at−80^°^C until processing.

Reverted nurses were obtained according to previously established methods [3]. Briefly, approximately 2000 foragers from the same colony were collected in front of the nest entrance with a bee vacuum, then transferred into a new nucleus hive with only two frames of worker larvae (1–3 day-old) and a queen. This new hive was then moved more than 7 kilometers away from the parent colony. 48 hours later, workers with their heads and thoraces in a cell containing a larva were considered as reverted nurses and were collected [3].

Twenty nurses, twenty foragers and twenty reverted nurses (three experimental groups) were respectively sampled. For each experimental group, ten were used for the DGE sequencing experiment and ten for the MeDIP-seq experiment.

### Digital Gene Expression (DGE) Tag Profiling

#### RNA isolation and digital transcriptomics

RNA library construction and deep sequencing were preformed by the BGI (Beijing Genomics Institute at Shenzhen, China). Total RNA was extracted from heads of nurses, foragers and reverted nurses respectively using TRIzol reagent (Invitrogen, Carlsbad, CA, USA) according to the manufacturer’s protocol. For each experimental group, 10 bee heads were pooled for each sample for gene expression analysis.

RNA quality was assessed by an Agilent 2100 Bioanalyzer (Agilent Technologies, Palo Alto, CA, USA). Sequencing libraries were constructed using Illumina Gene Expression Sample Prep Kit according to the manufacturer’s instructions. Briefly, 6 µg of total RNA was mixed with Sera-magnetic oligo beads to isolate mRNA. Oligo was used as primer to synthesize the first and second-strand of cDNAs. The bead-bound cDNA was subsequently digested with restriction enzyme NlaIII, which recognized and cut at CATG sites. Magnetic bead precipitation was used to purify digested cDNA fragments with 3′ ends, and the Illumina adaptor 1 was ligated to the sticky 5′ end of the digested bead-bound cDNA fragments. MmeI, recognized the junction of Illumina adaptor 1 and CATG site, and cut at 17 bp downstream of the CATG site, producing 21 bp tags with adaptor 1. These tags were subsequently ligated to Illumina adaptor 2 to generate tag libraries containing different adaptors at both ends. The cDNA tags were enriched with a PCR amplification of 15 cycles. The generated fragments were purified on a 6% TBE PAGE Gel. Double-strand cDNA fragments were denatured, and the resulting single-stranded molecules were fixed onto the Illumina sequencing chip (flowcell) for sequencing.

#### Analysis and mapping of DGE tags

Clean tags were obtained by filtering raw data to remove adaptor tags, low quality tags and single copy tags. These clean tags were deposited in the NCBI sequence read archive (SRX273353 for nurses, SRX273373 for foragers and SRX273936 for reverted nurses). All these clean tags were annotated using a database provided by Illumina. A preprocessed database of all possible CATG+17-nt tag sequences was created, using the honey bee genome (Amel 4.5) [12] (ftp://ftp.ncbi.nih.gov/genomes/Apis_mellifera/) and *Apis mellifera* transcriptome (OGS 1) (ftp.ncbi.nih.gov/genomes/*Apis_mellifera*/RNA/rna.fa.gz) data [12]. All the clean tags were aligned to the reference tag database, and only unambiguous tags were annotated. Based on the copy number in the library, the clean tags and clean distinct tags were classified. Sequencing saturation analysis of the library was performed. The number of unambiguously mapped clean tags for each gene was counted, then normalized to transcripts per million clean tags (TPM) to obtain normalized gene expression according to previously described methods [24], [25].

#### Identification of differentially expressed genes (DEGs)

We applied a rigorous statistical algorithm to identify DEGs across nurses, foragers and reverted nurses [26]. The formula to calculate the probability of a specific gene being expressed equally between two samples was defined as.
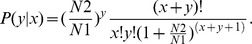
Where N1 and N2 indicate the total number of clean tags in sample 1 and sample 2, respectively, and x and y indicate the mapped clean tag counts of the transcript in each sample respectively. Then, the FDR (False Discovery Rate) method was applied to determine the threshold of the P-value in multiple tests. In this study, ‘FDR<0.001’, P-value<0.001 and the absolute value of log2-fold change >1 were used as the threshold criteria to define the significant differences in gene expression for each gene [27]. A user-written program to implement the above formula in R (R Core Team, Vienna, Austria) was employed for these statistical analyses. Among those significantly DEGs between forager/nurse and reverted nurse/forager comparisons, genes common to both lists but showing opposing directions of expression difference (for example upregulated in foragers relative to nurses (a forager/nurse comparison) and downregulated in reverted nurses relative to foragers (a reverted nurse/forager comparison)) were screened and annotated with Gene Ontology (GO), and functionally annotated with KEGG Orthology (KO) enrichment analysis. GO enrichment analysis of functional significance applies a hypergeometric test to map all differentially expressed genes to terms in GO database, looking for significantly enriched GO terms in differentially expressed genes compared to the complete genome. The formula is:



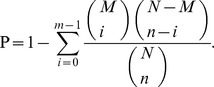
Where *N* is the number of all genes with a GO annotation in the bee genome; *n* is the number of those *N* genes differentially expressed; *M* is the number of all genes that are annotated to the certain GO terms; *m* is the number of differentially expressed genes in *M*. The hypergeometric test was also performed by a user-written program in R (R Core Team, Vienna, Austria).

KEGG pathway enrichment analysis identifies significantly enriched metabolic pathways or signal transduction pathways in differentially expressed genes comparing with the whole genome background. The calculating formula and the applied program were the same as that of GO analysis. Then, FDR (Q-value)≤0.05 was used as the threshold to determine the most significantly enriched pathways in DEGs.

#### Real-time quantitative PCR

To validate the sequencing results, six genes identified as highly and significantly differentially expressed between forager/nurse and reverted nurse/forager comparisons were chosen for confirmation of expression differences with real-time PCR. Real-time PCR primers were designed on the basis of the nucleotide sequence of the six chosen genes using Primer 5.0 software. Primer sequences and genes are summarized in Table S1 in File S1. Bees sampled for real-time PCR assays were from the same colony as that used for the DGE experiments. Total RNA was extracted from heads of nurse, forager and reverted nurse bees respectively using TRIzol reagent (Invitrogen, Carlsbad, CA, USA) according to the manufacturer’s protocol. The purity of the total RNA was determined as the 260 nm/280 nm ratio with expected values between 1.8 and 2.0. The RNA concentration of each RNA sample was measured in triplicate using a spectrophotometer (GeneQuant, Pharmacia). RNA integrity was determined by agarose gel (1.5%), electrophoresis, and ethidium bromide staining. The amount of RNA sample was standardized to 1 µg/µl for cDNA synthesis. cDNA was synthesized using MLV reverse transcriptase (Takara, Japan) according to the manufacturer’s instructions.

The cycling parameters were as follows: preliminary 94°C for 2 min, 40 cycles including 94°C for 15 sec, 58°C for 30 sec, and 72°C for 30 sec. The specificity of the PCR products was verified by melting curve analysis for each sample. Real-time PCR was carried out on each cDNA and analyzed in triplicate, after which the average threshold cycle was calculated. The relative expression levels were calculated using the formula reported by Liu and Saint [28]. β-actin was used as an appropriate internal control [29]. Relative expression levels were square root transformed to normalize the data. Expression differences between sample groups were analyzed by analysis of variance (ANOVA) using StatView (v 5.01, USA).

### Methylated DNA Immunoprecipitation Sequencing

#### DNA preparation and MeDIP-seq

DNA from nurses, foragers and reverted nurses respectively was isolated using a Universal Genomic DNA Extraction Kit (TaKaRa, DV811A). About 50 ng/per sample of purified DNA was then sent to BGI for MeDIP-seq analysis by a Illumina HiSeq™ 2000 (Illumina Inc, CA, USA). Sequence reads were prepared according to the manufacturer’s protocols following specific methods in Li et al. [20].

#### MeDIP-Seq sequence alignments

49 bp sequencing reads were deposited in the NCBI sequence read archive (SRX277287 for nurses, SRX277288 for foragers and SRX277289 for reverted nurses) and were mapped onto the honey bee genome (*Apis mellifera* L.) reference sequence (Amel 4.5) [12] (ftp://ftp.ncbi.nih.gov/genomes/Apis_mellifera/) using the high-performance alignment software ‘maq’ (http://maq.sf.net). Only unique alignments with no more than 2 mismatches were included in the analysis. Model-based Analysis of ChIP-Seq (MACS) was used to scan the methylated peaks (obtained methylated DNA by immunoprecipitation) in the genome [30]. Subsequently, the regions of differential methylation (DMRs) with DNA methylation peaks were employed for differential DNA methylation analysis.

#### Differential DNA methylation

Methylated regions were deemed significantly differentially methylated across nurses, foragers and reverted nurses with a P-value<0.05, a false discovery rate (FDR)<0.05 and at least a 1.5 fold change in sequence counts. Significantly DMRs in the whole genebody (DMGs) common to comparisons between forager/nurse and reverted nurse/forager with opposing directions of methylation difference (for example up-methylated in foragers relative to nurses (a forager/nurse comparison) and down-methylated in reverted nurses relative to foragers (a reverted nurse/forager comparison)) were employed for GO analysis and KEGG pathway analysis. The analyses used were the same as that employed for DEG analysis.

### Comparative Analysis of Significantly DEGs and DMGs

The list of significantly DMGs in forager/nurse and reverted nurse/forager comparisons were compared to the list of significantly DEGs in forager/nurse and reverted nurse/forager comparisons.

## Results

### Digital Gene Expression (DGE) Library Sequencing

**Figure 1:**
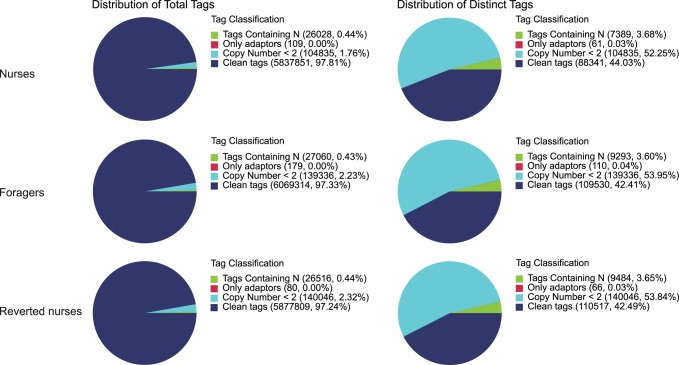
Distribution of total tags and distinct tags over different tag abundance categories in each sample. The numbers and percentage of tags containing N, empty tags with adaptor only, tags with copy number<2 and clean tags, are shown.

**Figure 2:**
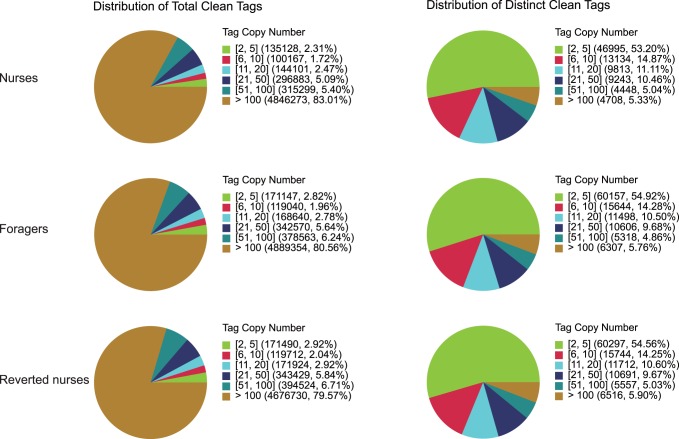
Distribution of total clean tags and distinct clean tags over different tag abundance categories in each sample. Numbers in the square brackets indicate the range of copy numbers for a specific category of tags.

Three DGE-tag libraries were generated from our experimental groups: nurses, foragers and reverted nurses. Table S2 in File S1 presents the number of raw tags, clean tags (after filtering out tags with evidence of sequencing errors) and tags that could be mapped to reference genes for samples of nurse, forager and reverted nurse. The percentage of clean tags relative to raw tags in each library is shown in Fig. 1. In each library, more than 79% of the identified clean tags occurred with copy numbers of more than 100, but these represented just 6% of the total diversity of tags (Fig. 2).

Saturation analysis was performed to check whether the total number of sequenced tags gave sufficient coverage of the expected number of distinct genes. As shown in Fig. S1 the number of newly detected genes stabilized at 2.5 M tags.

### Differentially Expressed Genes (DEGs) among Nurses, Foragers and Reverted Nurses

874 genes (from a total number of 7892 identified genes for nurses vs 8161 identified genes for foragers) were considered significantly differentially expressed between the nurse and forager samples (fold change≥2; FDR<0.001; P-value<0.001). Of these, 711 genes were up-regulated, and 163 genes were down-regulated in foragers compared with nurses (Table 1). 710 genes (from a total number of 8161 identified genes for foragers vs 8200 identified genes for reverted nurses) were significantly differentially expressed (fold change≥2; FDR<0.001; P-value<0.001) between foragers and reverted nurses. There were 516 up-regulated and 194 down-regulated genes in the reverted nurses relative to foragers (Table 1).

**Table 1:** Significantly up- or down- regulated genes in forager/nurse and reverted nurse/forager comparisons.

	Foragers relativeto nurses	Reverted nurses relativeto foragers	Foragers relative to bothnurses and reverted nurses	Common between forager/nurse andreverted nurse/forager lists
Up-regulated numberof genes	711	516	141	141 in foragers relative to both nursesand reverted nurses
Down-regulated numberof genes	163	194	88	−
Total number of DEGs	874	710	229	232
P-value	0.0023	1E-4	−	−

Of these DEGs, 232 genes were common to nurse/forager and reverted nurse/forager lists, regardless of their directions of gene expression change (Table 1). 229 genes exhibited reversed direction of gene expression change between forager/nurse and reverted nurse/forager comparisons: 141 of these genes were up-regulated in foragers relative to nurses but down-regulated in reverted nurses relative to foragers, 88 genes were down-regulated in foragers relative to nurses but upregulated in reverted nurses relative to foragers (Table 1). Pair-wise MANOVA was performed using R (R Core Team, Vienna, Austria) to analyse the observed and expected genes for the up-regulated and down-regulated genes. The P values were determined by a 10000-time permutation test. The results showed that the number of observed genes was significantly larger than that of expected by chance (*P<*0.01).

By comparing these 229 genes with a recent study [11], 147 genes (64.2%) showed the same trend in expression difference between nurses (including reverted nurses in our results) and foragers in both studies, while 39 genes (17%) presented opposite trends in expression, and 43 genes (18.8%) did not appear as differentially expressed in the earlier study (Table 2, Table S3 in File S1). The concordance between our list of DEGs and that of Liu et al. [11] was significantly greater than would have been expected by chance (chi-square contingency table analysis, χ^2^>50, *P<*0.0001). Further, 13 genes from the 147 genes were identified as significantly differentially expressed in both studies, and with the same direction of expression difference (Table S4 in File S1). These 13 genes included major royal jelly proteins (*MRJPs*), blue-sensitive opsin (*BLOP*), alpha-glucosidase (*Hbg3*), odorant-binding protein 4 (*Obp4*).

**Table 2:** Common significantly DEGs compared with Liu et al. and Whitfield et al.

	Adult head (Liu et al. 2011)	Adult brain (Whitfield et al.2003)
Entirely match	147	42
Opposite match	39	25
Ambiguous match	−	13
No information	43	149
Total	229	

An additional comparison with gene expression analysis using microarray data from Whitfield et al. [4] was also performed. Whitfield et al. [4] presented multiple comparisons of nurse and forager bees that included young nurse and old forager comparisons as well as age-matched nurse and forager samples taken from a single cohort colony. 42 out of 229 genes identified in this study (18.3%) were entirely consistent with all the gene lists of Whitfield et al. with the same direction of gene expression differences, regardless of young nurses and old foragers or single-cohort-colony nurses and foragers. 25 genes (10.9%) were expressed in an opposite way, 13 genes (5.7%) in an ambiguous way (similar direction of gene expression difference in some of Whitfield’s comparisons but not in others) and 149 genes (65.1%) did not appear as differentially expressed in Whitfield’s gene lists (Table 2, Table S3 in File S1). Through chi-square test, we found that the concordance of our DEG lists with those of Whitfield et al. [4] was significantly greater than expected by chance (chi-square contingency table analysis, χ^2^>15, *P<*0.001).

### Functional Classification of DEGs Identified as Significantly Differentially Expressed in both Forager/Nurse and Reverted Nurse/Forager Comparisons

We analyzed the 229 significantly DEGs common to forager/nurse and reverted nurse/forager comparisons with opposing directions of gene expression difference (Table S3 in File S1) in functional GO and KEGG. According to the GO terms, the 229 identified DEGs harbored 364 functional groups (Table S5 in File S1), and all these functional groups were restricted to three main categories (biological process, cellular component and molecular function). 70 genes were significantly enriched (Q-value <0.05) for the cellular component category, and of these 16 (22.9%) were annotated as active in the term of ribonucleoprotein complex (Table S6 in File S1). 104 genes were significantly enriched (Q-value<0.05) for the molecular function category, and of these, 13 (12.5%) were annotated as involved in the term of structural molecule activity (Table S6 in File S1). In addition, 97 genes with 4 terms were significantly enriched (Q-value<0.05) for the biological process category (Table S6 in File S1).

Through KEGG pathway analysis, we found that the 229 significantly DEGs were involved in 151 pathways (Table S7 in File S1), including drug metabolism, retinol metabolism, steroid hormone biosynthesis, ribosome, peroxisome proliferator-activated receptors (PPAR), the mammalian target of rapamycin (mTOR) and insulin/insulin-like (IIS) signaling pathway etc.

### Confirmation of DGE Data Using Real-time Quantitative PCR

**Figure 3:**
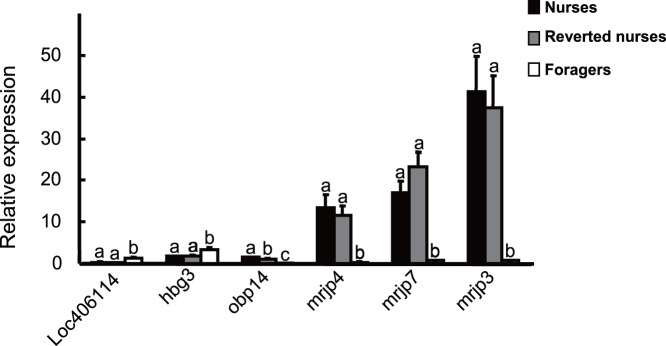
Verification of six differentially expressed genes between forager/nurse and reverted nurse/forager comparisons by quantitative RT-PCR. Different letters on top of bars indicate significant difference (P<0.05) with Fisher’s Protected Least Significant Difference. Each bar corresponds to a single group represented as the mean ± S.E. of its biological replicates. All six genes showed significant differences in gene expression level between forager/nurse and reverted nurse/forager comparisons by t-test.

In order to validate the sequencing results, six nursing- and foraging- related genes were selected for real-time quantitative PCR analysis (Fig. 3); The result showed that the real-time PCR results of all these genes were consistent with the Solexa expression data.

### Global Mapping of DNA Methylation

**Figure 4:**
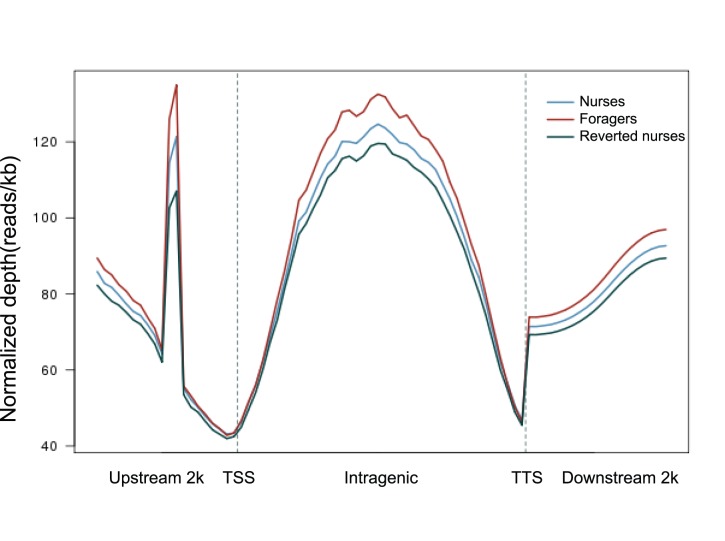
Distribution of reads around gene body. In upstream and downstream 2 kb regions, the regions were split into 20 equal regions. In the gene body, each gene was split into 40 equal regions. For each region, the normalized number of reads was calculated. “Y” axis is the average of normalized depth for each region.

From the MeDIP-seq analysis 20,299,343, 19,063,446 and 20,432,571 mapped reads were generated after filtration and quality checks in nurse, forager and reverted nurse samples, respectively. Over 77% of the reads were mapped and more than 72% of the reads were uniquely mapped to the honey bee genome (Table 3). Methylated peak regions covered about 14.54%, 14.24% and 14.94% of the genome in nurses, foragers and reverted nurses respectively. The distribution of DNA methylation in the 2 kb regions upstream of gene transcription start site (TSS), the intragenic regions, and the 2 kb regions downstream of gene transcription termination site (TTS) were shown in Fig. 4, and the component percentage of different repetitive sequence types were shown in Table S8 in File S1. The number of peak coverage on upstream 2 k, 5′-UTR, CDS, Intron, 3′-UTR and downstream 2 k for each sample can be seen in Table S9 in File S1.

**Table 3:** Summary of MeDIP-Seq Illumina GA data mapped to reference sequences.

Sample	Mapped reads	Percentage of mapped readin total reads (%)	Percentage of unique mappedreads (%)
Nurses	20,299,343	82.89	77.55
Foragers	19,063,446	77.84	72.75
Reverted nurses	20,432,571	83.43	78.25

### Differentially Methylated Genes (DMGs)

Distributions of up- or down- methylated genes among gene functional elements (including the whole genebody) in nurses, foragers and reverted nurses are presented in Table 4. Between nurse and forager libraries, a total of 366 significantly DMGs were detected, with 78 up-methylated genes and 288 down-methylated genes in foragers compared to nurses (fold change≥1.5; FDR<0.05; *P*<0.05) (Table 4). In comparisons of foragers and reverted nurses, 442 significantly DMGs were found, with 363 DMGs up-methylated in reverted nurses (fold change≥1.5; FDR<0.05; *P*<0.05) (Table 4). 165 DMGs were common to both the forager/nurse and reverted nurse/forager comparisons, with 33 DMGs up-methylated in foragers relative to nurses but down-methylated in reverted nurses relative to foragers, and 132 DMGs down-methylated in foragers relative to nurses but up-methylated in reverted nurses relative to foragers (Table S10 in File S1). We compared all these DMGs with a recent study from Herb et al. [16] which also compared methylation levels in nurse and forager bees. Only 10 genes were identified as differentially methylated between nurse and forager bees in both this study and that of Herb et al. [16], and even for these 10 genes the loci of methylation differed between the two studies (Table S11 in File S1).

**Table 4:** Differentially methylated genes on gene different element across three distinct workers.

gene elements	Foragers relative to nurses		Reverted nurses relative to foragers	
	down	up	down	up
genebody	288	78	79	363
Upstream 2 k	46	21	17	38
5′-UTR	48	25	26	56
CDS	112	52	63	136
Intron	217	63	64	273
3′-UTR	39	18	27	43
Downstream 2 k	49	27	38	62

### Functional Classification of Common Significantly DMGs

The 165 DMGs identified in both forager/nurse and reverted nurse/forager comparisons were assigned to terms in GO and KEGG databases. After the analysis of GO and KEGG, we found that several common DMGs were related to the calcium signaling pathway (*Itpr1*, *Pde1c* and *nAChRa6*), G-protein coupled receptor signaling pathway (*Dop3*), ecdysteroid signaling pathway (*E74*), regulation of actin cytoskeleton (*RhoGAPp190*), neuroactive ligand-receptor interaction (*mGlutR1*, *LOC726331*, and *LOC411632*), circadian rhythm (*tim2*), olfactory transduction (*LOC412801* and *Pde1c*), protein processing in endoplasmic reticulum (*Edem2*, *LOC100578248* and *LOC726410*), cell adhesion molecules (*Nrx-1*), and spliceosome (*LOC413509* and *LOC411632*) etc (Table S12 in File S1).

### Comparative Analysis of DEGs and DMGs

Comparing 874 significantly DEGs with 366 significantly DMGs between nurses and foragers, we found just 26 genes common to both lists, and while comparing 710 significantly DEGs with 442 significantly DMGs between foragers and reverted nurses, only 31 genes occurred on both lists. When comparing the 229 significantly DEGs that were common to forager/nurse and reverted nurse/forager comparisons with the 165 significantly DMGs that were common to forager/nurse and reverted nurse/forager comparisons, just 3 genes were common to both lists (Table 5).

**Table 5:** Comparison between significantly DMGs and DEGs in forager/nurse and reverted nurse/forager lists.

	Forager/nurse	Revertednurse/forager	Common between forager/nurseand reverted nurse/foragercomparisons	P-value
Significantly DEGs	874	710	229	4.720113e-12
Significantly DMGs	366	442	165	0
Overlapped genes	26	31	3	**−**
P-value	<0.0001	<0.0001	**−**	**−**

A cumulative hypergeometric test was performed to determine if the common genes (229 common significantly DEGs and 165 common significantly DMGs) were over- or under- represented. The result showed that the common genes were over-represented (*P*<0.0001). The canonical correlation tests were performed using the CANCORR procedure of SAS. Before the analysis, the data were converted into logarithms with base of 10 to make the data following a normal distribution. The first canonical correlation coefficient of the 26 common genes in the forager/nurse comparison is 0.9856 (*P*<0.0001), which explains 96.99% variation. The first canonical variables for foragers and nurses are a weighted difference of DEGs (0.0111 and −0.1301) and DMGs (1.6677 and 1.6810) respectively, with more emphasis on DMGs. The first canonical correlation coefficient of the 31 common genes in the reverted nurse/forager comparison is 0.9482 (*P*<0.0001), which explains 96.64% variation. The first canonical variables for reverted nurses and foragers are a weighted difference of DEGs (−0.0831 and −0.2614) and DMGs (0.9693 and 0.9907) respectively, with more emphasis on DMGs.

## Discussion

### Degree of Consistency of Gene Expression Differences between Nurse and Forager Bees within and between Studies

In this study 229 significantly DEG were common to the comparisons of forager/nurse and reverted nurse/forager, with opposing directions of gene expression difference. The degree of concordance between these gene lists was significantly greater than expected by chance (*P*<0.01). Furthermore, there was also a high degree of overlap between genes identified in this study and those identified by Liu et al. [11] (contingency table analysis, χ^2^>50, *P*<0.001). Specifically, most of *MRJPs* and *Rps* were consistently identified as up-regulated in nurses compared to foragers [11], [31]. Genes for odorant-binding proteins (*OBPs*), alpha-glucosidase (*Hbg3*), sodium channel protein paralytic (*Para*), blue-sensitive opsin (*BLOP*), the detoxification-associated genes (*CYP6AS4* and *CYP9Q1*) and inositol 1,4,5-triphosphate kinase (*IP3K*), and genes involved in the insulin/insulin-like signaling pathways (*IIS*), *mTOR* signaling pathway and *PPAR* pathway were found to be significantly more highly expressed in foragers than nurses in this and earlier studies [11], [32]–[39]. These results show that DGE analysis can yield reasonably repeatable findings across different studies.

However, we observed far less concordance with the gene list presented by Whitfield et al. [4]. This maybe because Whitfield et al. employed a microarray method rather than DGE to estimate gene expression, or, more likely because they analysed RNA samples from brains only, whereas our approach (in common with Liu et al. [11]) used RNA samples from whole heads. Our samples included the hypopharyngeal glands, which are locus for the synthesis of royal jelly proteins [1]. Thus it is reasonable that genes *MRJPs* were identified as significantly differentially expressed between nurses and foragers in our results whereas they were not identified in Whitfield’s study (Table S3 in File S1) since most *MRJP*s are not expressed in the brain.

Compared with microarrays, DGE analysis provides a wider coverage of the entire transcriptome [9]–[11]. Consequently, a few new genes detected in our results couldn’t be detected in Whitfield’s study (Table S3 in File S1). The high consistency between Liu’s and our studies, and a moderate consistency between Whitfield’s and our results suggest reasonable reliability to the DGE method when comparing the same tissues.

### DNA Methylation in Nurses, Foragers and Reverted Nurses

Our results confirmed that only a small fraction of the honeybee genome is methylated (the metylated peak regions covering about 14% of the genome in each sample) [13], in comparison to heavily methylated mammalian genomes [40]. Our results also confirmed that in honey bees most methylation occurred within CDS regions (Table S9 in File S1) [13], [14], [41], [42] and that honey bees have very low levels of methylation in in ALUs and transposons (Table S8 in File S1) [43].

A very recent study [16] suggested that not only were changes in DNA-methylation involved in the nurse/forager transition, but that some of these changes were reversible if forager bees reverted back to nursing [16]. Although our study used a different method to assess DNA methylation, we confirmed this conclusion (Table S10 in File S1). We found that 165 genes changed their methylation state with the nurse-forager transition, and after foragers reverted to nurses these methylations changes were also reversed. This confirms that there is a link between reversible DNA methylation changes and behavioural changes in honey bees. However, there was almost no overlap between the genes identified as differentially methylated in this study and that of Herb et al. [16]. One possible reason for this difference is because different tissues were used in the two studies: central brains were used in their research [16], while heads were used in ours. Heads included brains, eyes and glandular tissues, and this explains the reason why we obtained 643 DMGs across nurses, foragers and reverted nurses in total while they got 205 DMGs [16].

### Comparative Analysis of DEGs and DMGs

So far no study has directly compared the results of DGE and MeDIP-seq as tools for gene discovery in behavioural genomics. The results shown in Table 5 indicate a low level of ovelap between DEGs and DMGs. In fact, previous studies in human, chimpanzee and mice tissues and cell lines conducting association analysis for gene expression and DNA methylation have also shown a low overlap [44]–[46], some of which might be due to the limitation of techniques [45], or the complexity of the regulation of gene expression [44]–[46]. The technique of MeDIP microarray used in the previous study [45] might not be sensitive enough to detect all methylated regions since MeDIP mainly precipitates very highly methylated DNA fractions. Therefore, subsequent microarray analyses may mainly focus on certain peak regions close to the gene promoters, potentially missing many methylated domains beyond this region. Thus, the same situation might occur in our study, although MeDIP-seq instead of MeDIP microarray was used in the present study. Further, gene expression can be controlled by many other factors in addition to DNA methylation, such as chromatin state including histone marks and nucleosome positioning, transcription factor binding, and regulation by small RNAs. In light of this, the absence of overlap between DMG and DEG lists in our results is not surprising. It is possible that most of these DMGs were differentially spliced rather than differentially expressed, and most DEGs may not be regulated by direct methylation modification.

### General Conclusions

For the first time we report a comparison between gene expression with DNA methylation at a genome-wide level from nurses, foragers and reverted nurses. Our results confirmed that both gene expression and DNA methylation are involved in division of labour in workers. This study provides the first evidence that the overlap rate between gene expression and DNA methylation is low. Our study has produced novel insights into the mechanisms of task switching by a comparison between significantly differentially expressed and methylated genes between nurses and foragers.

## Supporting Information

Figure S1 Saturation analysis of clean tags. With the increase of total sequence number, the number of detected genes stabilized at 2.5 M.
(EPS)

File S1 **Contains: Table S1** Primers used for quantitative RT-PCR analysis. **Table S2.** Statistics of DGE sequencing. **Table S3.** Details for common significantly DEGs compared with Liu et al. and Whitfield et al. **Table S4.** Significantly DEGs common to the common gene lists and Liu’s with the same direction. **Table S5.** Gene Ontology assignments of common significantly DEGs between forager/nurse and reverted nurse/forager comparisons. These results were summarized in three main categories: cellular component, molecular function and biological process. The down regulated genes in foragers relative to nurses are the same as the up regulated genes in reverted nurses relative to foragers and vice versa. **Table S6.** Gene Ontology enrichment analysis of common significantly DEGs between forager/nurse and reverted nurse/forager comparisons. These results were summarized in three main categories: cellular component, molecular function and biological process. Terms from the cellular component, molecular function and biological process ontology with a p-value lower than 0.05. **Table S7.** The pathway analysis of up or down-regulated genes in common differentially expressed genes between forager/nurse and reverted nurse/forager comparisons. The down regulated genes in foragers relative to nurses are the same as the up regulated genes in reverted nurses relative to foragers and vice versa. **Table S8.** The component percentage of uniquely mapped reads in different repeat types. **Table S9.** Summary of peak coverage on gene elements. **Table S10.** Common significantly DMGs lists between forager/nurse and reverted nurse/forager comparisons. **Table S11.** Significantly DMGs lists overlapped with Herb et al. **Table S12.** GO and KEGG annotations about common significantly DMGs between forager/nurse and reverted nurse/forager comparisons.
(ZIP)
