# Supplementary material for: A Comparison of Digital Gene Expression Profiling and Methyl DNA Immunoprecipitation as Methods for Gene Discovery in Honeybee (Apis mellifera) Behavioural Genomic Analyses
Source: PLoS One. 2013 Sep 9;8(9):e73628. doi: 10.1371/journal.pone.0073628 (PMC3767799; doi:10.1371/journal.pone.0073628)
Supplement: File S1 — Contains: Table S1 Primers used for quantitative RT-PCR analysis. Table S2. Statistics of DGE sequencing. Table S3. Details for common significantly DEGs compared with Liu et al. and Whitfield et al. Table S4. Significantly DEGs common to the common gene lists and Liu’s with the same direction. Table S5. Gene Ontology assignments of common significantly DEGs between forager/nurse and reverted nurse/forager comparisons. These results were summarized in three main categories: cellular component, molecular function and biological process. The down regulated genes in foragers relative to nurses are the same as the up regulated genes in reverted nurses relative to foragers and vice versa. Table S6. Gene Ontology enrichment analysis of common significantly DEGs between forager/nurse and reverted nurse/forager comparisons. These results were summarized in three main categories: cellular component, molecular function and biological process. Terms from the cellular component, molecular function and biological process ontology with a p-value lower than 0.05. Table S7. The pathway analysis of up or down-regulated genes in common differentially expressed genes between forager/nurse and reverted nurse/forager comparisons. The down regulated genes in foragers relative to nurses are the same as the up regulated genes in reverted nurses relative to foragers and vice versa. Table S8. The component percentage of uniquely mapped reads in different repeat types. Table S9. Summary of peak coverage on gene elements. Table S10. Common significantly DMGs lists between forager/nurse and reverted nurse/forager comparisons. Table S11. Significantly DMGs lists overlapped with Herb et al. Table S12. GO and KEGG annotations about common significantly DMGs between forager/nurse and reverted nurse/forager comparisons. (ZIP) [file pone.0073628.s002.zip › Supporting information/Table S6.doc]

Table S6. Gene Ontology enrichment analysis of common significantly DEGs between forager/nurse and reverted nurse/forager comparisons. These results were summarized in three main categories: cellular component, molecular function and biological process. Terms from the cellular component, molecular function and biological process ontology with a p-value lower than 0.05.

| Terms from the cellular component ontology with a p-value lower than 0.05. | | | |
| --- | --- | --- | --- |
| **Gene Ontology term** | **Cluster frequency** | **Genome frequency of use** | **Corrected P-value** |
| [**ribonucleoprotein complex**](http://amigo.geneontology.org/cgi-bin/amigo/go.cgi?action=query&view=query&query=GO:0030529&search_constraint=terms) | **16 out of 70 genes, 22.9%** | **279 out of 3478 genes, 8.0%** | **0.00507** |
| Terms from the molecular function ontology with a P-value lower than 0.05. | | | |
| **Gene Ontology term** | **Cluster frequency** | **Genome frequency of use** | **Corrected P-value** |
| [**structural molecule activity**](http://amigo.geneontology.org/cgi-bin/amigo/go.cgi?action=query&view=query&query=GO:0005198&search_constraint=terms) | **13 out of 104 genes, 12.5%** | **165 out of 4501 genes, 3.7%** | **0.00675** |
| Terms from the biological process ontology with a P-value lower than 0.05. | | | |
| **Gene Ontology term** | **Cluster frequency** | **Genome frequency of use** | **Corrected P-value** |
| [**response to bacterium**](http://amigo.geneontology.org/cgi-bin/amigo/go.cgi?action=query&view=query&query=GO:0009617&search_constraint=terms) | **7 out of 97 genes, 7.2%** | **21 out of 4283 genes, 0.5%** | **5.11e-05** |
| [**defense response to bacterium**](http://amigo.geneontology.org/cgi-bin/amigo/go.cgi?action=query&view=query&query=GO:0042742&search_constraint=terms) | **6 out of 97 genes, 6.2%** | **20 out of 4283 genes, 0.5%** | **0.00080** |
| [**response to other organism**](http://amigo.geneontology.org/cgi-bin/amigo/go.cgi?action=query&view=query&query=GO:0051707&search_constraint=terms) | **7 out of 97 genes, 7.2%** | **39 out of 4283 genes, 0.9%** | **0.00484** |
| [**response to biotic stimulus**](http://amigo.geneontology.org/cgi-bin/amigo/go.cgi?action=query&view=query&query=GO:0009607&search_constraint=terms) | **7 out of 97 genes, 7.2%** | **41 out of 4283 genes, 1.0%** | **0.00682** |
