# Supplementary material for: A Comparison of Digital Gene Expression Profiling and Methyl DNA Immunoprecipitation as Methods for Gene Discovery in Honeybee (Apis mellifera) Behavioural Genomic Analyses
Source: PLoS One. 2013 Sep 9;8(9):e73628. doi: 10.1371/journal.pone.0073628 (PMC3767799; doi:10.1371/journal.pone.0073628)
Supplement: File S1 — Contains: Table S1 Primers used for quantitative RT-PCR analysis. Table S2. Statistics of DGE sequencing. Table S3. Details for common significantly DEGs compared with Liu et al. and Whitfield et al. Table S4. Significantly DEGs common to the common gene lists and Liu’s with the same direction. Table S5. Gene Ontology assignments of common significantly DEGs between forager/nurse and reverted nurse/forager comparisons. These results were summarized in three main categories: cellular component, molecular function and biological process. The down regulated genes in foragers relative to nurses are the same as the up regulated genes in reverted nurses relative to foragers and vice versa. Table S6. Gene Ontology enrichment analysis of common significantly DEGs between forager/nurse and reverted nurse/forager comparisons. These results were summarized in three main categories: cellular component, molecular function and biological process. Terms from the cellular component, molecular function and biological process ontology with a p-value lower than 0.05. Table S7. The pathway analysis of up or down-regulated genes in common differentially expressed genes between forager/nurse and reverted nurse/forager comparisons. The down regulated genes in foragers relative to nurses are the same as the up regulated genes in reverted nurses relative to foragers and vice versa. Table S8. The component percentage of uniquely mapped reads in different repeat types. Table S9. Summary of peak coverage on gene elements. Table S10. Common significantly DMGs lists between forager/nurse and reverted nurse/forager comparisons. Table S11. Significantly DMGs lists overlapped with Herb et al. Table S12. GO and KEGG annotations about common significantly DMGs between forager/nurse and reverted nurse/forager comparisons. (ZIP) [file pone.0073628.s002.zip › Supporting information/Table S7.doc]

**Table S7.** The pathway analysis of up or down-regulated genes in common differentially expressed genes between forager/nurse and reverted nurse/forager comparisons. The down regulated genes in foragers relative to nurses are the same as the up regulated genes in reverted nurses relative to foragers and [vice](app:ds:vice) [versa](app:ds:versa).

| Path_ID | Pathway | Q value | UP_regulated_genes in foragers relative to nurses  DOWN_regulated_genes in reverted nurses relative to foragers | DOWN_regulated_genes in foragers relative to nurses  UP_regulated_genes in reverted nurses relative to foragers |
| --- | --- | --- | --- | --- |
| ko00983 | Drug metabolism-other enzymes | 1.25E-06 | LOC409801;CYP6BE1;CYP6AS17;LOC409304;LOC409203;LOC411223;LOC409173;CYP6AS4;CYP6AS15;LOC725953;LOC727593;CYP6AS13;CYP6AQ1;CYP9Q1 | - |
| ko00980 | Metabolism of xenobiotics by cytochrome P450 | 1.54E-05 | GstD1;CYP6BE1;CYP6AS17;LOC409304;LOC409203;CYP6AS4;CYP6AS15;LOC727593;CYP6AS13;CYP6AQ1;CYP9Q1 | - |
| ko00982 | Drug metabolism - cytochrome P450 | 1.54E-05 | GstD1;CYP6BE1;CYP6AS17;LOC409304;LOC409203;CYP6AS4;CYP6AS15;LOC727593;CYP6AS13;CYP6AQ1;CYP9Q1 | - |
| ko00140 | Steroid hormone biosynthesis | 1.91E-05 | CYP6BE1;CYP6AS17;LOC409304;LOC409203;CYP6AS4;CYP6AS15;LOC727593;CYP6AS13;CYP6AQ1;CYP9Q1 | - |
| ko04976 | Bile secretion | 5.07E-05 | Nhe2;CYP6BE1;CYP6AS17;SCR-B2;LOC409304;LOC409203;CYP6AS4;CYP6AS15;LOC727593;SCR-B10;CYP6AS13;CYP6AQ1;CYP9Q1 | - |
| ko00830 | Retinol metabolism | 5.07E-05 | CYP6BE1;CYP6AS17;LOC409304;LOC409203;CYP6AS4;CYP6AS15;LOC727593;CYP6AS13;CYP6AQ1;CYP9Q1 | - |
| ko03010 | Ribosome | 1.62E-04 | - | RpS3;RpL10Ab;RpS13;RpL4;RpS26;RpL30;RpL29;RpL10;RpLP0;RpS15;RpL15;LOC550651 |
| ko01100 | Metabolic pathways | 3.10E-04 | GstD1;CYP6BE1;Ipp;LOC411609;CYP6AS17;LOC409304;LOC411978;Syt1;LOC410993;LOC408989;LOC726040;LOC409203;LOC727146;LOC724648;Fucta;LOC411223;LOC409173;CYP6AS4;LOC412810;LOC409709;LOC550785;LOC726362;LOC406114;CYP6AS15;LOC551268;LOC408787;LOC725953;LOC409768; | Aats-gln;Hex70a;LOC726625;Cbs;LOC408734;LOC409708;gi\|328783451\|ref\|XM_003250244.1\|;Cryl1;LOC412610 |
| ko00591 | Linoleic acid metabolism | 5.99E-03 | CYP6BE1;CYP6AS17;CYP6AS4;CYP6AS15;CYP6AS13;CYP6AQ1;CYP9Q1 | - |
| ko00053 | Ascorbate and aldarate metabolism | 1.35E-02 | LOC409304;LOC409203;LOC408650;LOC727593 | - |
| ko00740 | Riboflavin metabolism | 2.50E-02 | LOC726737;PPO | Hex70a;LOC725215 |
| ko00514 | Other types of O-glycan biosynthesis | 2.92E-02 | LOC551420;LOC409304;LOC409203;LOC408787;LOC727593 | - |
| ko00500 | Starch and sucrose metabolism | 3.32E-02 | LOC409304;LOC409203;LOC406114;LOC727593;Hbg3 | - |
| ko00562 | Inositol phosphate metabolism | 3.47E-02 | Ipp;Syt1;Fucta;LOC408650;LOC413817;Ip3k | - |
| ko00130 | Ubiquinone and other terpenoid-quinone biosynthesis | 4.16E-02 | LOC726040;LOC726362 | LOC726625 |
| ko00040 | Pentose and glucuronate interconversions | 4.51E-02 | LOC409304;LOC409203;LOC727593 | Cryl1 |
| ko04975 | Fat digestion and absorption | 7.76E-02 | LOC411609;SCR-B2;LOC551268;LOC410829;SCR-B10 | - |
| ko04142 | Lysosome | 7.90E-02 | LOC725038;LOC727146;LOC724761;LOC412810;LOC409709;LOC726737 | LOC409708;LOC725215 |
| ko04960 | Aldosterone-regulated sodium reabsorption | 7.90E-02 | LOC726045;Vkr;rl;LOC410732 | - |
| ko05215 | Prostate cancer | 8.15E-02 | GstD1;Vkr;LOC412150;rl;LOC410732 | - |
| ko03320 | PPAR signaling pathway | 9.47E-02 | LOC408989;LOC551268;LOC410732;LOC412353;LOC412166 | - |
| ko00600 | Sphingolipid metabolism | 1.16E-01 | LOC727146;LOC409709 | LOC409708 |
| ko00360 | Phenylalanine metabolism | 1.16E-01 | LOC726040;LOC726362 | LOC726625 |
| ko05160 | Insulin signaling pathway | 1.82E-01 | SCR-B2;LOC725803;LOC725870;rl;SCR-B10 | - |
| ko04978 | Mineral absorption | 2.76E-01 | Nhe2;gi\|328778297\|ref\|XR_119849.1\| | LOC551217 |
| ko00511 | Other glycan degradation | 2.76E-01 | LOC409709 | LOC409708 |
| ko00860 | Porphyrin and chlorophyll metabolism | 2.76E-01 | LOC409304;LOC409203;LOC727593 | - |
| ko04070 | Phosphatidylinositol signaling system | 2.98E-01 | Ipp;Syt1;Fucta;LOC413817;Ip3k | - |
| ko04672 | Intestinal immune network for IgA production | 3.35E-01 | - | LOC725946 |
| ko05216 | Thyroid cancer | 3.35E-01 | rl;LOC409061 | - |
| ko04013 | MAPK signaling pathway - fly | 3.87E-01 | rl | LOC726228 |
| ko04920 | Adipocytokine signaling pathway | 4.14E-01 | LOC726147;LOC727130;LOC408805 | - |
| ko00563 | Glycosylphosphatidylinositol(GPI)-anchor biosynthesis | 4.58E-01 | LOC724648;LOC411058 | - |
| ko04916 | Melanogenesis | 4.64E-01 | LOC411704;rl;PPO | Hex70a |
| ko00030 | Pentose phosphate pathway | 4.80E-01 | LOC550785;LOC409768 | - |
| ko04930 | Type II diabetes mellitus | 4.80E-01 | Vkr;rl | - |
| ko05218 | Melanoma | 4.98E-01 | Vkr;rl | - |
| ko04360 | Axon guidance | 5.24E-01 | LOC725803;LOC411704;LOC725870;rl | LOC725588 |
| ko04977 | Vitamin digestion and absorption | 5.24E-01 | LOC411609;SCR-B2;SCR-B10 | - |
| ko00260 | Glycine, serine and threonine metabolism | 5.31E-01 | GMCOX3;LOC409768 | Cbs |
| ko04620 | Toll-like receptor signaling pathway | 5.56E-01 | LOC725803;LOC725870;rl | - |
| ko00350 | Tyrosine metabolism | 5.82E-01 | GstD1;PPO | Hex70a |
| ko00052 | Galactose metabolism | 5.82E-01 | LOC411978;Hbg3 | - |
| ko00561 | Glycerolipid metabolism | 5.82E-01 | LOC411609;LOC408989;LOC551268 | - |
| ko00970 | Aminoacyl-tRNA biosynthesis | 5.82E-01 | LOC408787 | Aats-gln;LOC410806 |
| ko00533 | Glycosaminoglycan biosynthesis - keratan sulfate | 5.82E-01 | LOC727358 | - |
| ko04730 | Long-term depression | 5.82E-01 | Vkr;LOC411704;rl | - |
| ko05223 | Non-small cell lung cancer | 5.82E-01 | rl;LOC410732 | - |
| ko05213 | Endometrial cancer | 5.82E-01 | rl;LOC410732 | - |
| ko00051 | Fructose and mannose metabolism | 5.82E-01 | LOC550785;Pfrx | - |
| ko00601 | Glycosphingolipid biosynthesis - lacto and neolacto series | 5.97E-01 | LOC727358 | - |
| ko04146 | Peroxisome | 5.97E-01 | LOC410993;LOC411983;LOC551241 | Sod2 |
| ko04141 | Protein processing in endoplasmic reticulum | 5.97E-01 | LOC412150;Ero1L;Hsc70-3;LOC724341 | LOC551353;LOC552295 |
| ko04973 | Carbohydrate digestion and absorption | 5.97E-01 | LOC411978;LOC406114 | - |
| ko05212 | Pancreatic cancer | 5.97E-01 | rl;Rgl | - |
| ko05210 | Colorectal cancer | 6.16E-01 | rl;Rgl | - |
| ko04640 | Hematopoietic cell lineage | 6.16E-01 | Tspan1 | LOC725946 |
| ko04725 | Cholinergic synapse | 6.16E-01 | LOC411704;nAChRa6;rl | - |
| ko05140 | Leishmaniasis | 6.21E-01 | rl | LOC725946 |
| ko05200 | Pathways in cancer | 6.23E-01 | GstD1;Vkr;LOC412150;rl;Rgl;LOC409061 | LOC725588 |
| ko04020 | Calcium signaling pathway | 6.25E-01 | Fucta;nAChRa6;LOC413817;Ip3k | - |
| ko02010 | ABC transporters | 6.31E-01 | LOC412748;LOC410829 | - |
| ko04330 | Notch signaling pathway | 6.41E-01 | gi\|328790527\|ref\|XM_003251380.1\|;LOC411175 | - |
| ko04145 | Phagosome | 6.42E-01 | SCR-B2;LOC724761;LOC412810;SCR-B10 | CTL5 |
| ko04914 | Progesterone-mediated oocyte maturation | 6.48E-01 | Vkr;LOC411704;rl | - |
| ko04150 | mTOR signaling pathway | 6.51E-01 | rl;LOC410732 | - |
| ko00480 | Glutathione metabolism | 6.60E-01 | GstD1 | LOC412610 |
| ko04512 | ECM-receptor interaction | 6.70E-01 | LOC413739 | LOC725946;LOC411186 |
| ko05142 | Chagas disease (American trypanosomiasis) | 6.70E-01 | LOC411704;rl | - |
| ko05220 | Chronic myeloid leukemia | 6.70E-01 | rl | LOC725588 |
| ko05152 | Tuberculosis | 6.70E-01 | LOC724761;LOC412810;rl | CTL5 |
| ko05020 | Prion diseases | 6.70E-01 | rl;Hsc70-3 | - |
| ko04621 | NOD-like receptor signaling pathway | 6.70E-01 | LOC412150;rl | - |
| ko04520 | Adherens junction | 6.70E-01 | Vkr;Chd64;rl | - |
| ko05133 | Pertussis | 6.70E-01 | LOC411704;rl | - |
| ko05322 | Systemic lupus erythematosus | 6.70E-01 | - | LOC725308;LOC725344 |
| ko05120 | Epithelial cell signaling in Helicobacter pylori infection | 6.70E-01 | LOC410609;LOC412810 | - |
| ko04350 | TGF-beta signaling pathway | 6.70E-01 | LOC726045;rl | - |
| ko05214 | Glioma | 6.70E-01 | Vkr;rl | - |
| ko00280 | Valine, leucine and isoleucine degradation | 6.77E-01 | Syt1 | gi\|328783451\|ref\|XM_003250244.1\| |
| ko05219 | Bladder cancer | 7.59E-01 | rl | - |
| ko03018 | RNA degradation | 7.74E-01 | LOC409280;LOC411492 | LOC412602 |
| ko04320 | Dorso-ventral axis formation | 7.83E-01 | rl | LOC412319 |
| ko04514 | Cell adhesion molecules (CAMs) | 7.92E-01 | - | LOC725946;CTL5 |
| ko04612 | Antigen processing and presentation | 7.92E-01 | Hsc70-3 | - |
| ko01040 | Biosynthesis of unsaturated fatty acids | 8.05E-01 | LOC412166 | - |
| ko04966 | Collecting duct acid secretion | 8.17E-01 | LOC412810 | - |
| ko00520 | Amino sugar and nucleotide sugar metabolism | 8.29E-01 | - | LOC725646;LOC552683 |
| ko05323 | Rheumatoid arthritis | 8.39E-01 | LOC412810 | - |
| ko04540 | Gap junction | 8.39E-01 | LOC411704;rl | - |
| ko03060 | Protein export | 8.39E-01 | Hsc70-3 | - |
| ko05145 | Toxoplasmosis | 8.65E-01 | LOC411704;rl | - |
| ko04623 | Cytosolic DNA-sensing pathway | 8.65E-01 | Syt1 | - |
| ko05221 | Acute myeloid leukemia | 8.65E-01 | rl | - |
| ko04964 | Proximal tubule bicarbonate reclamation | 8.65E-01 | Nhe2 | - |
| ko04910 | Insulin signaling pathway | 8.65E-01 | Vkr;rl;LOC410732 | - |
| ko00590 | Arachidonic acid metabolism | 8.65E-01 | LOC410993 | - |
| ko04144 | Endocytosis | 8.65E-01 | LOC726045;Vkr;LOC409061 | LOC725588 |
| ko03040 | Spliceosome | 8.65E-01 | gi\|328790527\|ref\|XM_003251380.1\|;LOC551620 | LOC413280;LOC411091 |
| ko00071 | Fatty acid metabolism | 8.65E-01 | - | gi\|328783451\|ref\|XM_003250244.1\| |
| ko04724 | Glutamatergic synapse | 8.65E-01 | LOC411704;rl | - |
| ko00270 | Cysteine and methionine metabolism | 8.92E-01 | - | Cbs |
| ko05010 | Alzheimer's disease | 8.92E-01 | LOC410609;LOC551268;rl | LOC408734 |
| ko00020 | Citrate cycle (TCA cycle) | 8.96E-01 | - | LOC408734 |
| ko04340 | Hedgehog signaling pathway | 8.96E-01 | - | verm |
| ko03050 | Proteasome | 8.96E-01 | - | LOC726160 |
| ko03020 | RNA polymerase | 8.96E-01 | Syt1 | - |
| ko04115 | p53 signaling pathway | 8.96E-01 | - | LOC409207 |
| ko04670 | Leukocyte transendothelial migration | 8.96E-01 | LOC411704 | LOC725946 |
| ko04510 | Focal adhesion | 8.96E-01 | Vkr;rl;LOC410732 | LOC725946 |
| ko04650 | Natural killer cell mediated cytotoxicity | 8.96E-01 | rl | - |
| ko04380 | Osteoclast differentiation | 8.96E-01 | rl | - |
| ko04972 | Pancreatic secretion | 9.11E-01 | LOC411609;LOC406114;LOC551268 | - |
| ko00010 | Glycolysis / Gluconeogenesis | 9.11E-01 | LOC550785 | - |
| ko04974 | Protein digestion and absorption | 9.11E-01 | Nhe2;LOC552477 | - |
| ko04062 | Chemokine signaling pathway | 9.11E-01 | LOC411704;rl | - |
| ko03022 | Basal transcription factors | 9.11E-01 | - | LOC725906 |
| ko04114 | Oocyte meiosis | 9.11E-01 | Vkr;rl | - |
| ko04664 | Fc epsilon RI signaling pathway | 9.14E-01 | rl | - |
| ko04662 | B cell receptor signaling pathway | 9.22E-01 | rl | - |
| ko05211 | Renal cell carcinoma | 9.23E-01 | rl | - |
| ko04010 | MAPK signaling pathway | 9.23E-01 | rl;gi\|328781958\|ref\|XM_003250017.1\|;Nf1 | - |
| ko04660 | T cell receptor signaling pathway | 9.23E-01 | rl | - |
| ko00190 | Oxidative phosphorylation | 9.23E-01 | LOC412810 | LOC408734 |
| ko05164 | Influenza A | 9.23E-01 | LOC725803;LOC725870;rl | - |
| ko04310 | Wnt signaling pathway | 9.34E-01 | LOC411175 | verm |
| ko04370 | VEGF signaling pathway | 9.36E-01 | rl | - |
| ko04012 | ErbB signaling pathway | 9.38E-01 | rl | - |
| ko05412 | Arrhythmogenic right ventricular cardiomyopathy (ARVC) | 9.38E-01 | - | LOC725946 |
| ko04720 | Long-term potentiation | 9.47E-01 | rl | - |
| ko04745 | Phototransduction - fly | 9.54E-01 | inaD | - |
| ko05110 | Vibrio cholerae infection | 9.55E-01 | LOC412810 | - |
| ko04080 | Neuroactive ligand-receptor interaction | 9.64E-01 | nAChRa6 | Gr10 |
| ko04970 | Salivary secretion | 9.64E-01 | LOC406114 | - |
| ko04912 | GnRH signaling pathway | 9.64E-01 | rl | - |
| ko03015 | mRNA surveillance pathway | 9.64E-01 | - | LOC412602 |
| ko04722 | Neurotrophin signaling pathway | 9.64E-01 | rl | - |
| ko04810 | Regulation of actin cytoskeleton | 9.64E-01 | rl | Moe;LOC725946 |
| ko04120 | Ubiquitin mediated proteolysis | 9.64E-01 | LOC410838;LOC726045 | - |
| ko04971 | Gastric acid secretion | 9.64E-01 | LOC411704 | - |
| ko05131 | Shigellosis | 9.64E-01 | rl | - |
| ko04666 | Fc gamma R-mediated phagocytosis | 9.67E-01 | rl | - |
| ko05410 | Hypertrophic cardiomyopathy (HCM) | 9.85E-01 | - | LOC725946 |
| ko00240 | Pyrimidine metabolism | 9.85E-01 | Syt1 | - |
| ko05414 | Dilated cardiomyopathy | 9.85E-01 | - | LOC725946 |
| ko05012 | Parkinson's disease | 9.85E-01 | - | LOC408734 |
| ko05016 | Huntington's disease | 9.85E-01 | - | LOC408734;Sod2 |
| ko04270 | Vascular smooth muscle contraction | 9.89E-01 | rl | - |
| ko00230 | Purine metabolism | 9.89E-01 | Syt1 | - |
| ko04530 | Tight junction | 9.89E-01 | LOC411704 | - |
| ko03013 | RNA transport | 9.89E-01 | - | LOC412602 |
